# Long-Term Feeding with Curcumin Affects the Growth, Antioxidant Capacity, Immune Status, Tissue Histoarchitecture, Immune Expression of Proinflammatory Cytokines, and Apoptosis Indicators in Nile Tilapia, *Oreochromis niloticus*

**DOI:** 10.3390/antiox11050937

**Published:** 2022-05-10

**Authors:** Shimaa A. Amer, Doaa A. El-Araby, Haitham Tartor, Mahmoud Farahat, Nehal I. A. Goda, Mohamed F. M. Farag, Esraa M. Fahmy, Aziza M. Hassan, Mohamed F. Abo El-Maati, Ali Osman

**Affiliations:** 1 Department of Nutrition & Clinical Nutrition, Faculty of Veterinary Medicine, Zagazig University, Zagazig 44511, Egypt; drmahmoud3d@yahoo.com; 2 Department of Fish Health and Management, Central Laboratory for Aquaculture Research (CLAR), Agriculture Research Center, Abbassa, Abo-Hammad, Sharkia 44662, Egypt; dr.doaae@gmail.com; 3 Department of Fish Health and Welfare, Norwegian Veterinary Institute, Sentrum, P.O. Box 750, 0106 Oslo, Norway; haitham.tartor@vetinst.no; 4 Department of Histology and Cytology, Faculty of Veterinary Medicine, Zagazig University, Zagazig 44511, Egypt; nehalgoda@zu.edu.eg; 5 Department of Clinical Pathology, Faculty of Veterinary Medicine, Zagazig University, Zagazig 44511, Egypt; farag_cell@yahoo.com; 6 Department of Pharmacology, Faculty of Veterinary Medicine, Zagazig University, Zagazig 44511, Egypt; esraa.fahmy@zu.edu.eg; 7 Department of Biotechnology, College of Science, Taif University, P.O. Box 11099, Taif 21944, Saudi Arabia; a.hasn@tu.edu.sa; 8 Biochemistry Department, Faculty of Agriculture, Zagazig University, Zagazig 44511, Egypt; maboelmaati@gmail.com (M.F.A.E.-M.); aokhalil@zu.edu.eg (A.O.)

**Keywords:** Nile tilapia, curcumin, growth, histomorphology, disease resistance

## Abstract

The impact of dietary curcumin (CUR) on the growth, antioxidant activity, histomorphology of certain organs, proinflammatory cytokine production, and immune status of *Oreochromis niloticus* was evaluated. The fingerlings (*n* = 225, 41.60 ± 0.09 g/fish) were randomly allotted into five experimental groups in triplicate. Fish were fed basal diets complemented with 0, 200, 400, 600, or 800 mg curcumin/kg diet (CUR0, CUR200, CUR400, CUR600, and CUR800, respectively) for 10 weeks. An increase in fish growth was reported in the CUR200 and CUR400 groups. The feed conversion ratio was enhanced by 15% in the CUR400 group. Fish body protein content was increased in the CUR600 group (*p* ≤ 0.01). Body fat was decreased, and ash content was increased by CUR supplementation in a level-related way (*p* < 0.05). The villus height was increased in the CUR400 and CUR600 groups. The villus width was increased by CUR supplementation, with the best result found in the CUR600 group. The liver of CUR-fed fish displayed comparatively normal hepatocytes. TNF-α and caspase-3 were significantly upregulated by dietary CUR in a level-related way. The serum catalase activity and GSH level were increased in CUR200 and CUR400 groups. Curcumin supplementation boosted the serum SOD activity and reduced the MDA level. IL10 and IgM levels were increased in the CUR200 and CUR400 groups. Lysozyme activity was increased in the CUR200–400 groups. Serum complement 3 level was increased in the CUR400 group. The percentage survival of *O. niloticus* challenged with *Aeromonas hydrophila* was highest in the CUR200-CUR600 groups (100%) and decreased in the CUR800 group (80%). This study concluded that CUR could be added to Nile tilapia diets up to 400 mg·kg^−1^ to achieve better growth, antioxidant capacity, immune response, and intestinal histology. Long feeding periods on high levels of CUR (600 and 800 mg·kg^−1^) stimulate inflammatory reactions in fish tissues.

## 1. Introduction

Nile tilapia (*Oreochromis niloticus*) is an extensively raised freshwater fish [1]. Innovative tools and feeding strategies are needed to increase production volume and improve fish health [2,3,4,5]. The availability of high-quality and affordable feed in required quantities is essential to satisfy the upcoming needs for food from aquaculture [6]. The use of herbal additives in aquaculture has attracted the attention of many researchers worldwide [7,8,9]. The research shows the positive effects of using phytobiotics and herbal products in raising fish. It has been described to augment many end-points such as growth and feed consumption, play a role as an immune stimulant and antistress agent, and enhance the antimicrobial status of fish [10,11,12].

*Aeromonas hydrophila* bacterial infection is a common and impactful disease related to intensive rearing [13]. Antibiotics have often been applied to prevent bacterial diseases in aquaculture, leading to bacterial antibiotic resistance and residues in fish fillets [13,14]. Therefore, it is essential to discover environmentally friendly alternatives to antibiotics to prevent disease. Phytogenic products are appropriate for future growth and health promotions in aquaculture because there are no residual problems or adverse impacts on fish health, humans, and the ecosystem [10,12].

Curcumin is a yellow polyphenolic compound found in turmeric *Curcuma longa* L. rhizomes [15]. In aquaculture, curcumin has been shown to have significant biological actions as a growth enhancer [16], along with antioxidant [17], hepatoprotective [18], immune-enhancing [19], bactericidal [20], and antiparasitic effects [21]. Manju et al. [22] reported raised body protein content and reserved liver lipid peroxidation of curcumin-fed climbing perch (*Anabas testudineus*); this could have been the reason for decreased liver damage by carbon tetrachloride in Jian carp, *Cyprinus carpio* var. Jian [17], and reduced liver toxicity caused by aflatoxin B1 in Nile tilapia [23] by regulating antioxidant enzyme and nonenzymatic compounds. Therefore, dietetic CUR can improve liver health and function by raising its antioxidant ability. Curcumin has powerful antioxidant, antitumor, anti-inflammatory, and antidepressant activities [24,25]; thus, it is commonly used in clinical therapy. Curcumin has been reported to have a hepatoprotective effect [26]. Curcumin and its synthetic analog protect against the oxidative stress caused by thermally and alcohol oxidized sunflower oil [27]. Curcumin prevents liver damage caused by alcohol in mice via the NF-κB signaling pathway [28].

We hypothesized that long-term feeding with dietary curcumin might affect the growth, immune, and inflammatory status of fish. Thus, this study evaluated the effects of long-term feeding of different levels of curcumin on the growth, serum antioxidant capacity, histopathological integration of intestine and liver, immune status, and resistance to *Aeromonas hydrophila* infection in *Oreochromis niloticus*.

## 2. Material and Methods

### 2.1. Curcumin Isolation and Identification

Turmeric was purchased from the local market in Zagazig City, Egypt. Turmeric powder was extracted with 95% ethanol using the soxhlet apparatus. When the solvent was no longer colored orange, the extraction was terminated. The isolation was performed using column chromatography on a silica gel with a mobile phase of dichloromethane–methanol (97:3) described by Nurjanah and Saepudin [29]. Ten milligrams of purified pigment were diluted in 10 mL of HPLC-grade methanol at a 1 mg/mL concentration and filtered through a 0.45 µm filter (Millipore Corp., Milford, MA, USA). The resultant liquid was subjected to HPLC on a C18 column at a 1 mL/min flow rate as described in Naidu et al. [30]. A UV detector at 425 nm was used to detect curcumin, and the peak area in relation to the standard was used to calculate the concentration.

### 2.2. Fish and Experimental Design

The present study was conducted at the Central Laboratory for Aquaculture Research (CLAR), Abo-Hammad, Sharkia, Egypt. All procedures were performed on animals following Egyptian laws on animal experimentation, with the approval of the Egyptian Veterinary Authority, Ethical Committee for Animal Experiments (ZU-IACUC/2021).

Healthy *Oreochromis niloticus* fingerlings (*n* = 225; 41.60 ± 0.09 g/fish) were purchased from Abbassa Fish Hatchery, Sharkia Province, Egypt. The health status of the fingerlings was tested before the experimentation according to CCAC [31]. Fish were placed in 15 static glass aquariums (50 cm × 40 cm × 60 cm) and acclimated to laboratory conditions for 14 days before the experiment. Twenty-five percent of the tank water was changed daily, and a complete exchange of aquarium water was carried out twice a week. The water quality was kept within the advised limits during the experimental period, according to regulations [32]. All fish were manually fed the basal diet twice daily (at 9 a.m. and 2 p.m.) until satiety during the acclimation period.

After acclimation, the fish were randomly assigned into five experimental groups in triplicate (15 fish/replicate). For the next 10 weeks, each of the five experimental groups was fed a basal diet complemented with one concentration of curcumin (0, 200, 400, 600, or 800 mg/kg diet), and the groups are hereafter referred to as CUR0, CUR200, CUR400, CUR600, and CUR800, respectively. Diets were prepared in the form of 2 mm pellets and formulated in accordance with the NRC [33] (Table 1). Fish and diets were analyzed following the AOAC [34]. Fish were monitored daily for symptoms of illness or death.

### 2.3. Growth Performance Parameters

At the start of the experiment, initial fish body weights were documented, and final weights and consumed feed were recorded at the end of the trial. The subsequent growth performance parameters were determined in accordance with Castell and Tiews [35] as follows:

Total weight gain (g/fish) = WT − WI, where WT is the final weight (FW, g/fish), and WI is the initial weight (g/fish).
Average daily weight gain (ADWG) (g/fish/day) = total weight gain/experimental days.
Feed conversion ratio (FCR) = total feed intake (g)/total weight gain (g).
Specific growth rate (SGR) (%/day) = 100 × (ln WT − ln WI)/time in days, where ln is the natural logarithm.
Protein productive efficiency (PPE) = protein gain (g)/protein intake (g).
The protein efficiency ratio (PER) was calculated in accordance with Stuart and Hung [36] as PER = total gain (g)/protein intake (g).

### 2.4. Histology 

The effect of curcumin supplementation on the histological integrity of fish intestine and liver was investigated. At the end of the feeding trial, samples of the intestine and liver tissues were collected from each group (three fish per group) and prepared for routine histological examination following the steps described by Al-Khalaifah et al. [12].

### 2.5. Immunohistochemical Processing

At the end of the feeding trial, samples of intestine and liver were collected from each group (three fish per group) and used for immunohistochemical analysis of TNF-alpha and caspase-3 (TNF-alpha antibody: Cat. No. NBP2-61611 and caspase-3 antibody: Cat. No. NB600-1235, Novus Biologicals, Centennial, CO, USA) according to El-Araby et al. [37]. 

### 2.6. Sample Collection

At the end of the experiment, blood samples (nine fish per group) were collected without anticoagulants and left to coagulate for 15–20 min at 4 °C in the refrigerator before centrifugation for 15 min 3000 rpm. The separated serum was stored at −20 °C until further analysis of the biochemical and immunological parameters.

### 2.7. Clinico-Biochemical Analysis of the Blood

By using the colorimetric diagnostic kits of Spectrum-Bioscience (Egyptian Company for Biotechnology, Cairo, Egypt), the total cholesterol (TC) and triglycerides (TG) were measured following the methods of Allain et al. [38] and McGowan et al. [39], respectively. According to Vassault et al. [40], the high-density lipoprotein cholesterol (HDL-C) was measured using the enzymatic colorimetric method. The serum low-density lipoprotein cholesterol (LDL-C) level was measured following the Iranian formula LDL-C = TC/1.19 + TG/1.9 − HDL/1.1 − 38 [41]. The liver function tests (alanine aminotransferase (ALT) and aspartate aminotransferase (AST)) were estimated following Murray [42] and Burtis and Ashwood [43], respectively.

### 2.8. Immunological Indices

The effect of curcumin food supplementation on serum immunological indices was examined by comparing the serum levels of total IgM, complement component 3 (C3), and interleukin 10 (IL10), as well as serum lysozyme activity, between fish fed a diet supplemented with different concentrations of curcumin and those fed the control diet (basal diet only). MyBioSource Co. ELISA kits Cat. Nos. MBS282651, MBS005953, and Cat. No. MBS044038 were used to measure the serum IgM, C3, and IL10, respectively, according to the manufacturer’s instructions [37]. Lysozyme activity was assessed by spectrophotometry according to Ellis [44].

### 2.9. Serum Antioxidant Activity

We compared the serum levels of three antioxidant enzymes (catalase (CAT), superoxide dismutase (SOD), and reduced glutathione (GSH)) and one surrogate marker of oxidative stress (malondialdehyde (MDA)) between the five experimental groups to examine the effect of food supplemented with different curcumin concentrations on the antioxidant capacity of fish serum. Different ELISA kits were used for the determination of MDA level and CAT, SOD, and GSH activity (My Biosource Co. CAT NO. MBS2700234, MBS038818, MBS705758, and MBS2540412, respectively) following the manufacturer’s procedures for each kit [37].

### 2.10. Bacterial Challenge Test

At the end of the trial, fish from all groups were injected intraperitoneally with pathogenic *A. hydrophila* at a dosage of 0.1 mL of cell suspension containing 4 × 10^5^ cells/mL using McFarland standard tubes [45]. The isolate was previously isolated from dying fish at the Department of Fish Diseases and Management, Faculty of Veterinary Medicine, Zagazig University, and confirmed pathogenic for *O. niloticus*. Fish deaths and clinical symptoms were noted for 2 weeks following Lucky [46]. Fish mortalities were used to calculate the percentage of survival.

Percentage (%) survival = (number of fish in each group post bacterial challenge/number of fish before bacterial challenge) × 100.

### 2.11. Statistical Analysis

ANOVA was applied on the basis of polynomial orthogonal contrasts. Linear and quadratic regression equations were calculated using SPSS Version 17 for Windows (SPSS Inc., Chicago, IL, USA). Post hoc Tukey’s test was used to assess differences among means; the variation in the data was expressed as pooled SEM, and the significance level was set at *p* < 0.05.

## 3. Results

### 3.1. HPLC Analysis

**Figure 1:**
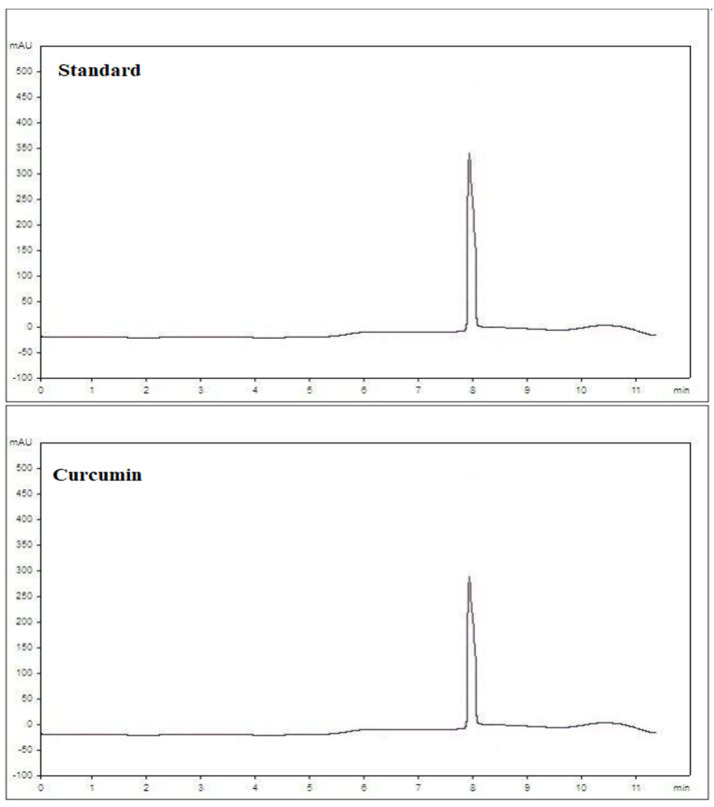
HPLC chromatogram of curcumin isolated from turmeric root compared to standard curcumin.

Curcumin identification was achieved using the C18 column; the chromatogram is presented in Figure 1 compared to the standard. The peak area in relation to the standard was used to calculate the concentration, and the concentration of curcumin was recorded as 15.2 µg/mL.

### 3.2. Growth Performance and Fish Whole-Body Composition

Quadratic increases in the FW, ADWG, TWG, and SGR were observed in the CUR200 and CUR400 groups compared to the CUR0 group (*p* < 0.05). The FW was raised by 15% and 18% in the CUR200 and CUR400 groups, respectively (*p* ≤ 0.01). The TWG was increased by 31% and 39% in the CUR200 and CUR400 groups, respectively (*p* ≤ 0.01). The SGR was improved by 21% and 27% in the CUR200 and CUR400 groups, respectively (*p* ≤ 0.01). The FCR was quadratically improved in CUR400 by 15% compared to the CUR0 group (*p* ≤ 0.01). The total feed intake, PER, and PPE did not differ significantly among CUR-supplemented groups compared to the control group (*p* > 0.05) (Table 2). 

The initial body composition was 23.4% dry matter, 56% crude protein, 29.6% ash, and 10.6% fat. There was no significant difference in the dry matter (DM) content among groups (*p* > 0.05). However, it was increased numerically in the CUR400 group. A linear increase in protein content was observed in the CUR600 group (*p* ≤ 0.01), while a linear decrease in fat content was found at a dose-dependent level (*p* ≤ 0.01). Ash content was raised by curcumin supplementation (*p* < 0.05) (Table 3).

### 3.3. Histopathological Findings

**Figure 2:**
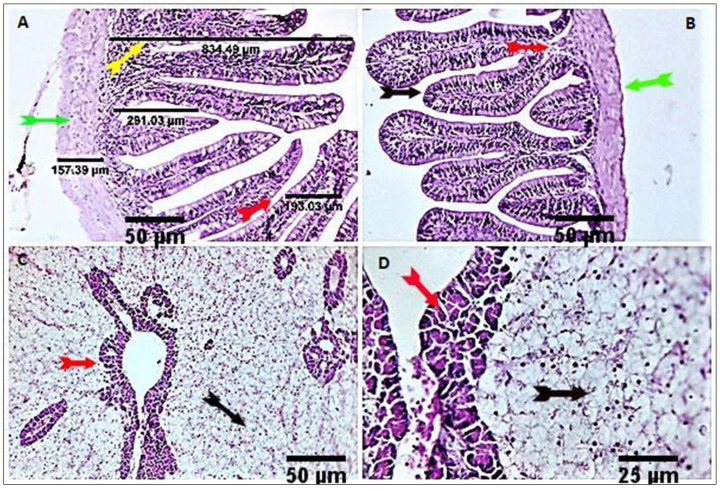
Stained sections of the anterior and posterior intestines of the control group showed normal histological structure (**A**,**B**) with variable numbers of goblet cells (yellow and red arrows). The liver of the control group showed normal histomorphological structures with normal features of hepatopancreatic structures (red arrows) and hepatic cord orientation (black arrows) (**C**,**D**). Scale bars = 25 µm, 50 µm.

**Figure 3:**
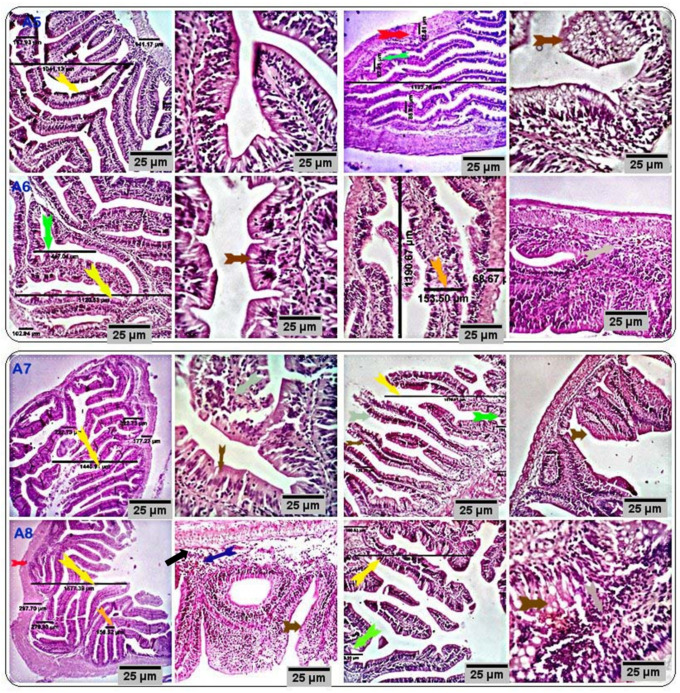
Stained sections of the anterior and posterior intestines of the respective treatment groups (CUR200, CUR400, CUR600, and CUR800) showed normal histomorphological structures with an increase in the villus height and crypt depth (yellow and green arrows), goblet cell number (brown arrows), and the number of mucosal and submucosal immune cells (black arrows). A moderate number of eosinophilic granular cells are seen in the submucosal tissue of all experimental groups, particularly groups CUR400 and CUR600 (blue arrow). Scale bars = 25 µm. (A5: CUR200, A6: CUR400, A7: CUR600, and A8: CUR800).

The anterior and posterior parts of the intestine of the control groups showed a normal histological structure (Figure 2). Anterior intestines of the respective treatment groups (CUR200, CUR400, CUR600, and CUR800) showed a normal histological structure with increased villus height (VH) and crypt depth (CD), goblet cell number, and immune cell number in the mucosa and submucosa (plasma cells, lymphocytes, and histiocytes). The only exception was a decreased thickness of the muscular coat in the CUR400 group compared with the control and other groups (Figure 3). The dimensions of the villus height, villus width, crypts length, and muscular layer thickness of the anterior intestines in the different treatments are shown in Table 4. Villus height was increased linearly and quadratically in the CUR400 and CUR600 groups compared to the CUR0 (*p* ≤ 0.01). Villus width was increased linearly and quadratically by curcumin supplementation, with the best result found in the CUR600 group (*p* ≤ 0.01). The crypt depth was linearly and quadratically increased by curcumin supplementation, with the highest result in the CUR600 group (*p* ≤ 0.01). However, it was decreased in the CUR400 group (*p* ≤ 0.01). The muscular layer thickness was reduced linearly and quadratically in the CUR200 and CUR400 groups and increased in the CUR800 group compared to the CUR0 group (*p* ≤ 0.01). Posterior intestines of the respective treatment groups (CUR200, CUR400, CUR600, and CUR800) showed normal histological structure with more pronounced goblet cells and immune cell numbers (lymphocytes, plasma cells, and histiocytes). A moderate number of eosinophilic granular cells were observed in the submucosal tissue of all experimental groups, particularly the CUR400 and CUR600 groups (Figure 3).

**Figure 4:**
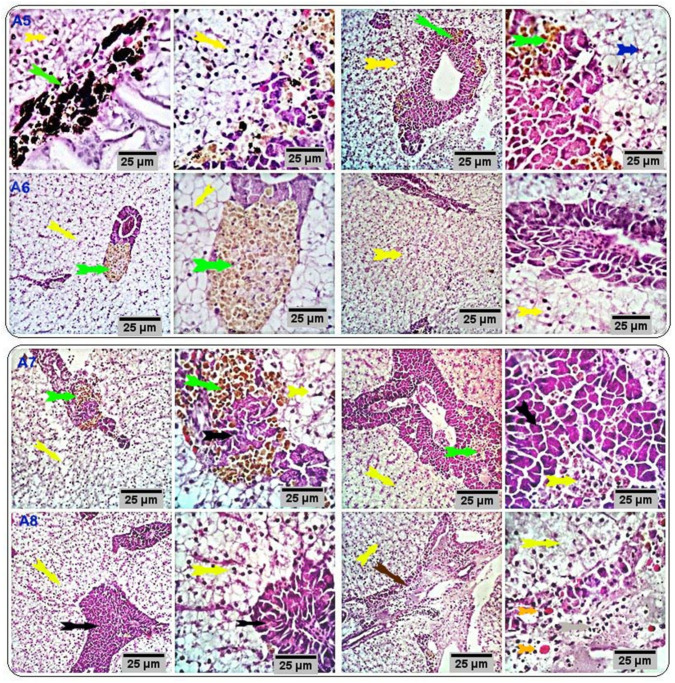
Stained sections of the liver of the respective treatment groups (CUR200, CUR400, CUR600, and CUR800) showing histomorphological structures comparable to that of the control group (yellow and black arrows) with activated hepatopancreatic acini and normal vascular structures with few surrounding numbers of melano-macrophages. Other sections showed marked infiltration of melano-macrophages, particularly at the hepatic portal/pancreas (green arrows) with occasional vascular dilation, edema, infiltration of eosinophilic granular cells (orange arrows), and pancreatic acinar disorganization and/or degeneration (brown arrow); some of the hepatocytes appeared degenerated (blue arrow). Scale bars = 25 µm. (A5: CUR200, A6: CUR400, A7: CUR600, and A8: CUR800).

Liver sections of the respective treatment groups (CUR200, CUR400, CUR600, and CUR800) showed normal histomorphological structures. However, some examined fishes showed comparatively normal hepatocytes, activated hepatopancreatic acini, and normal vascular structures with few surrounding melano-macrophages. Other fishes recorded marked infiltration of melano-macrophages, particularly at their hepatic portal/pancreas, with occasional vascular dilation, edema, infiltration of eosinophilic granular cells, and pancreatic acinar disorganization and or degeneration, particularly at high levels of curcumin; moreover, some of the hepatocytes were degenerated (Figure 2 and Figure 4).

### 3.4. Immunohistochemical Analysis

**Figure 5:**
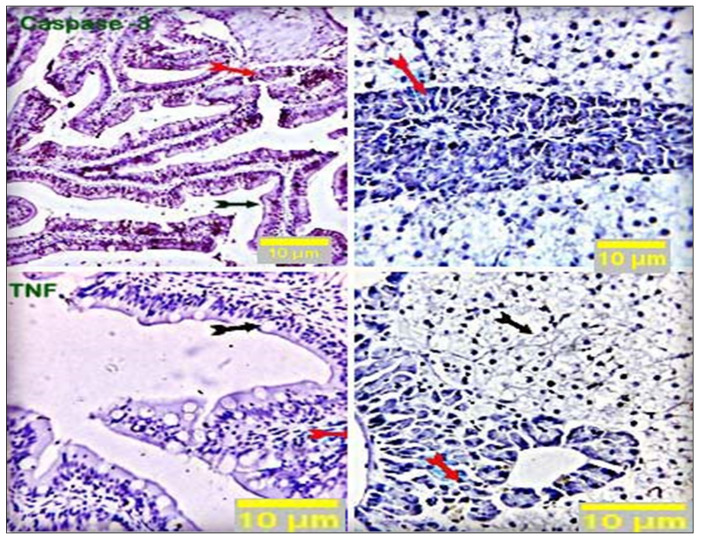
Stained sections of the intestine and liver of the control group showed negative staining reactions in all the examined parts of the intestinal and hepatic tissue (black and red arrows) against caspase-3 and TNF-α antibodies. Nearly 10% of hepatic portal cells reacted positively to caspase-3. Scale bars = 10 µm.

**Figure 6:**
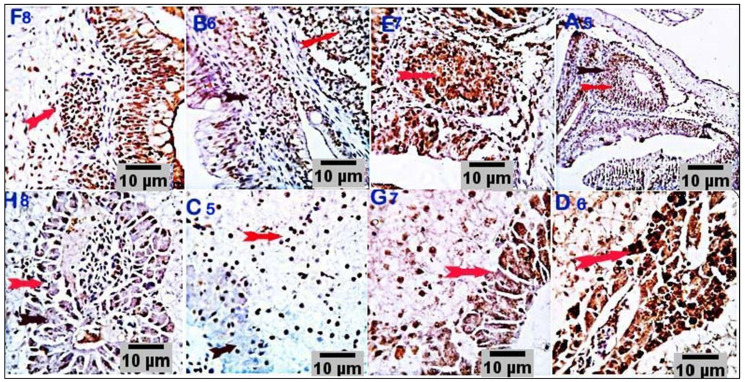
Stained sections of the intestine (F8, B6, E7, and A5) and liver (H8, C5, G7, and D6) of the respective treatment groups (CUR200, CUR400, CUR600, and CUR800) immunostained against caspase-3 showing a moderate number of caspase-positive cells with brownish cytoplasmic reactivity in intestinal cells of different experimental groups (25%, 32%, 35%, and 45% of the cells in CUR200, CUR400, CUR600, and CUR800 groups, respectively). Meanwhile, liver sections (C5, D6, G7, and H8) immunostained with anti-caspase-3 monoclonal antibody showed mild to moderate brownish stainabilities in a variable number of hepatic and hepatic portal pancreatic cells (1.5%, 17%, 18%, and 23% of the cells in CUR200, CUR400, CUR600, and CUR800 groups, respectively). Scale bars = 10 µm.

**Figure 7:**
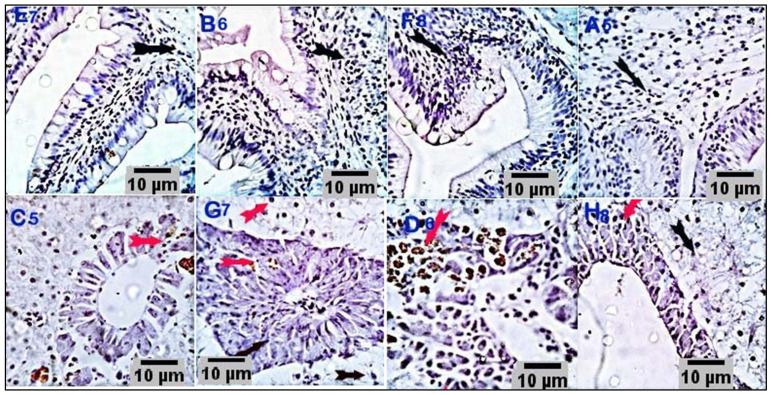
Stained sections of the intestine (E7, B6, F8, and A6) and liver (C5, G7, D6, and H8) of the respective treatment groups (CUR200, CUR400, CUR600, and CUR800, respectively) immunostained against TNF-α. Sections from intestines of different treatment groups (E7, B6, F8, and A6) immunostained against TNF-α, showing a weak positive reaction in a few mucosal and submucosal mononuclear cells (2.5%, 3.5%, 4%, and 14% of cells in CUR200, CUR400, CUR600, and CUR800 groups, respectively). Liver sections (C5, G7, D6, and H8) also demonstrated weak reactivities in a variable number of hepatic and hepatic portal cells (3.9%, 10%, 25%, and 25% of cells in CUR200, CUR400, CUR600, and CUR800 groups, respectively). Scale bars = 10 µm.

Immunostained sections from the control group showed negative staining reactions against caspase-3 and TNF-α antibodies in all the examined parts of the intestinal and hepatic tissues. Nearly 10% of hepatic portal cells reacted positively to caspase-3 (Figure 5). Intestinal sections from the respective treatment groups (CUR200, CUR400, CUR600, and CUR800) immunostained against caspase-3 showed moderate brownish cytoplasmic reactivity in a moderate number of cells in the respective experimental groups (25%, 32%, 35%, and 45% of the cells in CUR200, CUR400, CUR600, and CUR800 groups, respectively). Meanwhile, liver sections (CUR200, CUR400, CUR600, and CUR800) immunostained by anti-caspase-3 monoclonal antibody demonstrated mild to moderate brownish stainabilities in a variable number of hepatic and hepatic portal/pancreatic cells (1.5%, 17%, 18%, and 23% of the cells in CUR200, CUR400, CUR600, and CUR800 groups, respectively) (Figure 6). On the other hand, the intestinal sections from different treatment groups (CUR200, CUR400, CUR600, and CUR800) immunostained against TNF-α revealed a weak positive reaction in a few mucosal and submucosal mononuclear cells (2.5%, 3.5%, 4%, and 14% in CUR200, CUR400, CUR600, and CUR800 groups, respectively). Liver sections also demonstrated weak reactivities in a variable number of hepatic and hepatic portal cells (3.9%, 10%, 25%, and 25% of cells in CUR200, CUR400, CUR600, and CUR800 groups, respectively) (Figure 7).

### 3.5. Clinco-Biochemical Indices

Dietary curcumin did not significantly affect the serum levels of TC, TG, HDL-C, LDL-C, VLDL-C, AST, and ALT (*p* > 0.05) (Table 5).

### 3.6. Antioxidant Status

Quadratic increases in the serum catalase activity and GSH level were observed in the CUR200 and CUR400 groups; levels decreased in the CUR600 and CUR800 groups but remained higher than in the CUR0 group (*p* ≤ 0.01). A linear and quadratic increase in the serum SOD activity and a decreased MDA level were observed with CUR addition (*p <* 0.05) (Table 6).

### 3.7. Immunological Parameters

The serum levels of IL10 and IgM were quadratically increased in CUR200 and CUR400; levels decreased in CUR600 and CUR800 but remained significantly higher than in the CUR0 group (*p* ≤ 0.01). Lysozyme activity was linearly and quadratically increased in the CUR200–400 groups (*p =* 0.02, *p* ≤ 0.01, respectively). The serum complement 3 level was quadratically increased in the CUR400 group (*p* ≤ 0.01) (Table 6).

### 3.8. Bacterial Challenge

The highest survival rate of *O. niloticus* following *A. hydrophila* challenge was recorded in the CUR200–CUR600 groups (100%) and then decreased in the CUR800 group (80%) compared to the CUR0 group (10%).

## 4. Discussion

The current study showed improved growth performance by dietary addition of curcumin with the highest growth in the CUR200–400 groups. The lowest FCR was observed in the CUR400 group, which was improved by 15% related to the control non-supplemented group. The growth performance began to decrease in CUR600–800 groups to become non-significantly different from the control group, which may be due to the observed decrease in the fat content of the fish body upon increasing the level of curcumin supplementation. The increased growth after CUR addition could be due to the role of CUR in improving digestion through increased trypsin and lipase activities in the pancreas and intestine, as well as liver amylase activity, thereby resulting in faster growth [47]. Moreover, curcumin effectively increased the activities of the enzymes located at the gut brush border responsible for nutrient degradation and assimilation, thus improving nutrient availability and growth [47].

Curcumin may have digestive properties, thus improving growth performance. The absorption capacity of the intestine also affects nutrient utilization [48]. The improved growth may also be due to the improvement of most intestinal histomorphometric measures in curcumin-fed fish. Abd El-Hakim et al. [49] reported that CUR 200 mg·kg^−1^ supplementation improved the growth of *O. niloticus,* which was retarded by feeding on a diet contaminated with melamine. Sruthi et al. [16] reported that curcumin supplementation by 0.5% and 1% increased weight gain and SGR and improved FCR of *Oreochromis mossambicus,* which may be explained by the ability of curcumin to raise the activities of protease, lipase, and α-amylase enzymes in the intestine. They found that the activities of the digestive enzymes were elevated in fish fed a diet complemented with 0.5% and 1% curcumin compared with the non-supplemented group [50]. Curcumin enhances digestibility and nutrient utilization by decreasing nutrient excretion [51]. Results of the current study are consistent with those of Mahmoud et al. [52], who demonstrated that CUR supplementation increased body weight and weight gain of *O. niloticus* challenged with *Pseudomonas fluorescens*. Furthermore, CUR increased the growth performance of grass carp (*Ctenopharyngodon idells*) [53] and rainbow trout (*Oncorhynchus mykiss*) raised under various stocking densities [54]. Curcumin supplementation (20 g/kg) for 8 weeks significantly improved the growth and feed conversion ratios of rainbow trout above those fed the control diet [55]. Similarly, dietary curcumin at 5, 10, and 20 g/kg levels could improve feed efficiency and growth in Nile tilapia, *O. niloticus* [18,56].

The present study indicated that body protein content was raised in the CUR600 group. The body fat was decreased at a dose-dependent level, and the ash content was increased by curcumin supplementation. These results confirmed the results of growth performance where the body weight decreased upon increasing the CUR level due to a reduction in the body’s fat content. Abd El-Hakim et al. [49] demonstrated that dietary CUR (200 mg·kg^−1^) significantly increased the protein and lipid content of Nile tilapia muscles; this could have been due to the enhancing effect of curcumin on the intestinal microbiota and digestive enzyme activities of fish [47].

AST and ALT are prominent biomarkers of hepatic impairment [57]. The present study showed that serum AST and ALT levels were within the normal range, indicating that curcumin supplementation did not affect liver function. In addition, dietary curcumin did not affect the serum lipid profile. These results were supported by the results of liver histology that showed comparatively normal hepatocytes, activated hepatopancreatic acini, and normal vascular structures with surrounding small numbers of melano-macrophages in curcumin-fed groups. However, the highest level of CUR (800 mg/kg diet) showed some alterations in the liver represented by occasional vascular dilation, edema, infiltration of eosinophilic granular cells, and pancreatic acinar disorganization or degeneration. In the study of Cao et al. [17], dietary curcumin (0.1–0.5%) decreased AST and ALT levels and reduced hepatocyte degeneration induced by CCl4 injection in Jian carp (*Cyprinus carpio* var. Jian), indicating a hepatoprotective effect of curcumin through increasing the antioxidative activities and decreasing IL1b, NF-kB, TNF-α, and IL12 expression. Similarly, El-Barbary [56] reported the hepatoprotective effect of curcumin in aflatoxin B_1_-injected *Oreochromis niloticus*, with the best results observed at lower curcumin levels (10 g/kg diet) than at higher levels (20 g/kg diet). Furthermore, dietary curcumin (200 and 400 mg/kg) could reduce deltamethrin-induced liver damage in northern snakehead *(Channa argus*) through the Nrf2 and NF-κB signaling pathways [58].

TNF-α is a proinflammatory cytokine secreted from activated macrophages, vital in regulating innate immune functions and inflammatory responses [59]. Caspases are the primary mediators of programmed cell death (apoptosis). Between them, caspase-3 is a commonly activated death protease, which stimulates several vital cellular proteins [60]. In the present study, liver and intestinal immunostained sections from curcumin-fed fish revealed mild to moderate reactions against caspase-3 and weak positive reactions against TNF-α after a 10 week feeding period, particularly at high CUR levels. Our results suggested that long-term feeding on curcumin at high levels may stimulate inflammatory reactions in tissues and cell apoptosis, as indicated by the significant upregulation of caspase-3 and TNF-α. These results support the histological findings of the liver and suggest that the changes in the liver tissues may have been due to the stimulation of proinflammatory cytokines such as TNF-α and apoptosis markers such as caspase-3.

On the other hand, CUR was reported to significantly downregulate IL1β and TNF-α gene expression [49,61]. An earlier study reported that curcumin reduced the response of inflammatory cytokines such as chemokines, TNF-α, IL1, IL6, IL2, IL8, and IL12, through inhibition of NF-κB and TNF-α binding [62]. Significant anti-inflammatory effects were shown by CUR administration at levels of 100–200 mg/kg BW in a large variety of animal experiments [63].

Regarding the effect of dietary curcumin on the antioxidant activity of *O. niloticus*, the results revealed that the serum catalase activity and GSH level were increased in the CUR200 and CUR400 groups. Curcumin supplementation increased the serum SOD activity and decreased the MDA level. Similarly, Cao et al. [17] noted increased antioxidant activities in crucian carp (*Carassius carassius*) fed a diet fortified with curcumin. Moreover, CUR addition increased the mRNA expression of SOD and GPx in crucian carp (*Carassius carassius*) [47]. The transcription of antioxidant enzymes mainly produces the antioxidant effect of CUR through stimulation of the nuclear factor erythroid 2 signaling pathway, which is responsible for scavenging free radicals [64].

The innate immune system of fish is the main defense against pathogen attack and is more effective in fish than in mammals [65]. Several cellular components (e.g., leukocytes) and humoral components (e.g., C-reactive protein, complement, and lysozyme) deeply participate in the innate immune response [66]. In the present study, IL10, IgM, and lysozyme activity were raised in CUR200 and CUR400 groups. The serum complement 3 level was raised in the CUR400 group, indicating curcumin’s immunomodulatory effect. Yonar et al. [55] demonstrated that CUR supplementation could activate the humoral immunity in rainbow trout, marked by a significant increase in IgM and total protein values in curcumin-fed fish. Moreover, a marked rise in leukogram values was detected in curcumin-fed fish (200 mg/kg) [67]. The plasma lysozyme activity and immunoglobulin levels were significantly improved by dietary CUR in challenged rainbow trout (*Oncorhynchus mykiss*) [55] and Nile tilapia (*O. niloticus*) [19].

The essential function of the immune system is protecting against attacking pathogens. Therefore, the host resistance challenge test is a highly comprehensive immune response test of great biological importance as it assesses the collective immune reactions of the whole organism [68]. Moreover, the percentage of survival is one of the most apparent metrics for evaluating the immunological effect of a challenge test [69]. In the current study, *O. niloticus* was challenged with *A. hydrophila* following a 10-week feeding period. The survival rate of *O. niloticus* challenged with *A. hydrophila* was the highest in the CUR200–CUR600 groups (100%) and decreased in the CUR800 group (80%) compared to the CUR0 group (10%). Abd El-Hakim et al. [49] reported that CUR improved the disease resistance of the fish against bacteria during the challenge, which could be closely related to its immune activity noted here. Likewise, dietary CUR enhanced disease resistance to *A. hydrophila* in rohu (*Labeo rohita*) [70] and *O. niloticus* [19]. Baldissera et al. [71] noted that silver catfish (*Bagrus filamentosus*) fed dietary curcumin (150 mg/kg) acquired 100% disease resistance against *Streptococcus agalactiae*. Curcumin-fed rainbow trout (2%) challenged with *Aeromonas salmonicida* showed the highest relative percentage survival (RPS) (76.67%) compared with the control (36.67%) [55].

## 5. Conclusions

This study evaluated the potential impacts of graded dietary levels of curcumin (200–800 mg/kg diet) in *O. niloticus*. Results showed that curcumin could be included in Nile tilapia diets up to 400 mg·kg^−1^ for increased growth, antioxidant capacity, and immune responses. Curcumin supplementation increased intestinal histology markers and increased the disease resistance of *O. niloticus* against *Aeromonas hydrophiala*, with the most prominent effect shown at 200–600 mg/kg supplementation level. Long feeding periods on high levels of curcumin (600 and 800 mg·kg^−1^) are not recommended, however, as they cause changes in liver histology and stimulate inflammatory reactions and cell apoptosis through upregulation of caspase-3 and TNF-α.

## Figures and Tables

**Table 1:** Proximate chemical composition of the basal diet (g·kg^−1^ on a dry weight basis).

Ingredients	g·kg^−1^
Fish meal 70.7% CP	180
Yellow corn	216.5
Soybean meal 49% CP	275
Corn gluten 67% CP	70
Wheat flour	100
Wheat bran	60
Fish oil	65
Methionine	3.5
Vitamins and minerals mixture	30
Proximate composition (g·kg^−1^)	
CP	355.43
Fat	102.80
NFE ^1^	441.16
Crude fiber	36.62
Ash	63.97
Lysine	20.20
Methionine	10.72
GE MJ/kg ^2^	20.72

^1^ Nitrogen-free extract, determined by difference = 100 − (protein% + fat% + crude fiber% + ash%). ^2^ Gross energy (GE) was calculated according to the NRC (2011) as 23.6 kJ/g protein, 39.5 kJ/g lipids, and 17.0 kJ/g NFE. CP: crude protein.

**Table 2:** Effect of dietary curcumin (CUR) on the growth performance of *O. niloticus*.

	IBW (g/fish)	FBW (g/fish)	ADWG (g/fish)	TWG (g/fish)	TFI (g/fish)	FCR	PER	PPE	SGR
CUR0	41.8	81.2 ^b^	0.56 ^b^	39 ^b^	51	1.30 ^ab^	2.2	1.2	0.95 ^b^
CUR200	41.8	93.4 ^a^	0.74 ^a^	51.5 ^a^	64.7	1.26 ^ab^	2.2	1.2	1.15 ^a^
CUR400	41.3	96 ^a^	0.78 ^a^	54.7 ^a^	60	1.10 ^b^	2.3	1.3	1.20 ^a^
CUR600	41.4	81.7 ^b^	0.58 ^b^	40 ^b^	51.5	1.28 ^ab^	2.2	1.2	0.97 ^b^
CUR800	41.4	83 ^b^	0.59 ^b^	41.5 ^b^	58.7	1.41 ^a^	2	1.1	0.99 ^b^
SEM	1.46	2.18	0.03	2.18	1.95	0.03	0.04	0.02	0.03
Linear Reg. ^#^	0.14	0.18	0.22	0.22	0.80	0.16	0.31	0.53	0.32
Quadratic Reg. ^#^	0.51	≤0.01	≤0.01	≤0.01	0.14	≤0.01	0.13	0.12	≤0.01

^#^ The regressions were considered significant at *p* < 0.05. IBW, initial body weight; FBW, final body weight; ADWG, average daily weight gain; TWG, total body weight gain; TFI, total feed intake; FCR, feed conversion ratio; PER, protein efficiency ratio; PPE, protein productive efficiency; SGR, specific growth rate. Variation in the data was expressed as pooled SEM. ^a,b^ Mean values in the same column with different superscripts differ significantly (*p* < 0.05). CUR0, CUR200, CUR400, CUR600, and CUR800: basal diets supplemented with 0, 200, 400, 600, or 800 mg curcumin/kg diet, respectively.

**Table 3:** The effects of dietary curcumin (CUR) on the proximate whole-fish body composition of *O. niloticus*.

	DM % *	Crude Protein % **	Crude Lipids % **	Ash % **
CUR0	25.6	54.5 ^bc^	20 ^a^	21.4 ^b^
CUR200	23.5	53.9 ^c^	16.5 ^ab^	27.3 ^a^
CUR400	26.2	55.3 ^abc^	15 ^bc^	25.7 ^a^
CUR600	23.8	56.1 ^a^	13.00 ^bc^	28.8 ^a^
CUR800	22.7	55.9 ^ab^	11.50 ^c^	28.8 ^a^
SEM	0.49	0.25	0.96	0.94
Linear Reg. ^#^	0.04	≤0.01	≤0.01	≤0.01
Quadratic Reg. ^#^	0.26	0.73	0.55	0.02

^#^ The regressions were considered significant at *p* < 0.05. Variation in the data was expressed as pooled SEM. ^a,b,c^ Mean values in the same column with different superscripts differ significantly (*p* < 0.05). CUR0, CUR200, CUR400, CUR600, and CUR800: basal diets supplemented with 0, 200, 400, 600, or 800 mg curcumin/kg diet, respectively. * On a fresh basis; ** on a dry matter basis.

**Table 4:** The effects of dietary curcumin (CUR) on intestinal histomorphometric measures (µm) of *O. niloticus*.

	Villous Height	Villous Width	Crypt Depth	MT
CUR0	868 ^b^	97 ^d^	146 ^d^	162 ^b^
CUR200	1034 ^ab^	145 ^b^	223 ^b^	131 ^c^
CUR400	1178 ^a^	146.50 ^b^	129 ^e^	66 ^d^
CUR600	1203 ^a^	214 ^a^	313 ^a^	164 ^b^
CUR800	1062 ^ab^	124 ^c^	184 ^c^	198 ^a^
SEM	26.70	8.83	17.38	12.85
Linear Reg. ^#^	≤0.01	≤0.01	≤0.01	≤0.01
Quadratic Reg. ^#^	≤0.01	≤0.01	≤0.01	≤0.01

^#^ The regressions were considered significant at *p* < 0.05. Variation in the data was expressed as pooled SEM. ^a,b,c,d,e^ Mean values in the same column with different superscripts differ significantly (*p* < 0.05). MT: muscular layer thickness. CUR0, CUR200, CUR400, CUR600, and CUR800: basal diets supplemented with 0, 200, 400, 600, or 800 mg curcumin/kg diet, respectively.

**Table 5:** The effects of dietary curcumin (CUR) on the blood biochemical parameters of *O. niloticus*.

	TC (mg/dL)	TG (mg/dL)	HDL-C (mg/dL)	LDL-C (mg/dL)	VLDL-C (mg/dL)	AST (u/L)	ALT (u/L)
CUR0	184	171	50.8	99.5	34	66	4.8
CUR200	107	99	51.6	70	19.7	27.7	16
CUR400	189	137	51.9	109.	27	78	25
CUR600	204	186	50	116	37	48.8	34
CUR800	229	205	47	141	41	32.5	18.8
SEM	7.23	4.34	16.7	14.5	0.70	10.4	2.8
Linear Reg. ^#^	0.09	0.07	0.06	0.11	0.07	0.20	0.14
Quadratic Reg. ^#^	0.25	0.06	0.08	0.39	0.06	0.39	0.15

^#^ The regressions were considered significant at *p* < 0.05. Variation in the data was expressed as pooled SEM. Mean values in the same column with different superscripts differ significantly (*p* < 0.05). TC: total cholesterol, TG: triglycerides, HDL-C: high-density lipoprotein cholesterol, LDL-C: low-density lipoprotein cholesterol, ALT: alanine aminotransferase, AST: aspartate aminotransferase. CUR0, CUR200, CUR400, CUR600, and CUR800: basal diets supplemented with 0, 200, 400, 600, or 800 mg curcumin/kg diet, respectively.

**Table 6:** The effects of dietary curcumin on the antioxidant and immune status of *O. niloticus*.

	Antioxidant Activity				Immunological Indices			
	CAT (ng/mL)	GSH (ng/mL)	MDA (ng/mL)	SOD (ng/mL)	IL10 (pg/mL)	IgM (ng/mL)	Lysozymes (ng/mL)	C3 (mg/mL)
CUR0	1.3 ^c^	7 ^c^	20 ^a^	105 ^b^	157 ^b^	213 ^b^	5 ^b^	92.3 ^b^
CUR200	33.4 ^a^	24.6 ^ab^	7 ^b^	253 ^a^	238 ^a^	1835 ^a^	19.8 ^a^	127 ^ab^
CUR400	34.6 ^a^	25.9 ^a^	3.5 ^b^	270 ^a^	240 ^a^	2075 ^a^	20.4 ^a^	178 ^a^
CUR600	16.9 ^b^	16.2 ^b^	4.8 ^b^	229 ^a^	188 ^ab^	913 ^b^	20 ^a^	126 ^ab^
CUR800	16.7 ^b^	15.8 ^bc^	2.7 ^b^	219 ^a^	167 ^b^	386 ^b^	17.6 ^ab^	124.18 ^b^
SEM	4.17	2.29	2.17	20	12.29	254	2.2	9.7
Linear Reg. ^#^	0.14	0.12	≤0.01	≤0.01	0.44	0.21	0.02	0.08
Quadratic Reg. ^#^	≤0.01	≤0.01	≤0.01	≤0.01	≤0.01	≤0.01	≤0.01	≤0.01

^#^ The regressions were considered significant at *p* < 0.05. Variation in the data was expressed as pooled SEM. ^a,b,c^ Mean values in the same column with different superscripts differ significantly (*p* < 0.05). CAT: catalase, SOD: superoxide dismutase, GSH: reduced glutathione, MDA: malondialdehyde. C3: complement 3, IL10: interleukin 10. CUR0, CUR200, CUR400, CUR600, and CUR800: basal diets supplemented with 0, 200, 400, 600, or 800 mg curcumin/kg diet, respectively.

## Data Availability

The datasets used and analyzed during the current study are available from the corresponding author on reasonable request.
